# Proteoglycans/Glycosaminoglycans: From Basic Research to Clinical Practice

**DOI:** 10.1155/2014/295254

**Published:** 2014-12-21

**Authors:** George Tzanakakis, Ilona Kovalszky, Paraskevi Heldin, Dragana Nikitovic

**Affiliations:** ^1^Lab of Anatomy-Histology-Embryology, Medical School, University of Crete, 71003 Heraklion, Greece; ^2^1st Department of Pathology and Experimental Cancer Research, Semmelweis University, Budapest H-1085, Hungary; ^3^Ludwig Institute for Cancer Research, Matrix Biology, Department of Medical Biochemistry and Microbiology, Uppsala University, 751 05 Uppsala, Sweden

Extracellular matrices (ECM) represent a complex network of proteins and glycosaminoglycans (GAGs) constituting the cell microenvironment including elastin, laminins, fibronectin, and GAG decorated proteoglycans (PGs). PGs are composed of independent structural domains, the sequences and arrangements of which are highly conserved and discretely glycosylated, thus determining a varying degree of matrices organization. On the other hand, both bound GAG chains and free GAGs such as hyaluronan (HA) bestow voluminosity to the ECM, due to negative charges they carry and their subsequent water binding ability. Therefore these molecules participate in maintaining the bulk, shape, and strength of tissues in vivo. However, PGs/GAGs provide much more than just mechanical and structural support but are critically important for cell growth, survival, and differentiation [1, 2]. Moreover, these molecules are key participants of various disease processes including inflammation, atherosclerosis, autoimmune diseases, and cancer [3–5]. Importantly, GAG/PG effects are clearly dependent on the specific correlation among their abundance, distribution, and disease type/stage. The evidence that PGs/GAGs have a key role in various pathological conditions has led to the conclusion that understanding the changes in PG/GAG expression and fine structure that occur in disease may lead to opportunities to develop innovative and selective therapies. We invited authors to submit original research and review articles that seek to designate discrete roles for GAGs/PGs as diagnostic markers and/or therapy targets for specific disease types and grades. Therefore, the submissions for this special issue focused on the utilization of pluripotent characteristics of PGs/GAGs and their synthesizing and/or degrading enzymes in the ongoing battle against disease.

The topics discussed in depth by experts in the field and published in this special issue include the following: mechanisms of GAG/PG action and their potential application, development of novel disease markers, roles of PGs/GAGs in wound healing (therapeutical aspects), development of immunotherapeutic strategies involving GAGs/PGs, roles of PGs/GAGs in tissue engineering, potential application of PGs/GAGs in axon regeneration, roles of PGs/GAGs in cartilage regeneration, synthesis of reliable carriers specifically designed to deliver discrete GAGs/PGs to target disease tissues, utilization of GAGs/PGs as carriers for targeted therapy delivery.

We hope that readers will find that this special issue highlights the important advances that are presently being accomplished in the preclinical and clinical applications in the rapidly growing PG/GAG field. Hopefully, the highlighted advances will encourage the application of novel drug development for elevating disease states.

*George Tzanakakis* *Ilona Kovalszky* *Paraskevi Heldin* *Dragana Nikitovic*
